# *Schisandra chinensis* Fructus and Its Active Ingredients as Promising Resources for the Treatment of Neurological Diseases

**DOI:** 10.3390/ijms19071970

**Published:** 2018-07-06

**Authors:** Minyu Zhang, Liping Xu, Hongjun Yang

**Affiliations:** 1 School of Traditional Chinese Medicine, Capital Medical University, Beijing 100069, China; zhangminyu@ccmu.edu.cn (M.Z.); xulp@ccmu.edu.cn (L.X.); 2 Beijing Key Lab of TCM Collateral Disease Theory Research, Capital Medical University, Beijing 100069, China; 3 Institute of Chinese Materia Medica, China Academy of Chinese Medical Sciences, Beijing 100700, China

**Keywords:** *Schisandra chinensis* Fructus, active ingredients, neurological diseases, molecular mechanism

## Abstract

Neurological diseases (NDs) are a leading cause of death worldwide and tend to mainly affect people under the age of 50. High rates of premature death and disability caused by NDs undoubtedly constrain societal development. However, effective therapeutic drugs and methods are very limited. *Schisandra chinensis* Fructus (SCF) is the dry ripe fruit of *Schisandra chinensis* (Turcz.) Baill, which has been used in traditional Chinese medicine for thousands of years. Recent research has indicated that SCF and its active ingredients show a protective role in NDs, including cerebrovascular diseases, neurodegenerative diseases, or depression. The key neuroprotective mechanisms of SCF and its active ingredients have been demonstrated to include antioxidation, suppression of apoptosis, anti-inflammation, regulation of neurotransmitters, and modulation of brain-derived neurotrophic factor (BDNF) related pathways. This paper summarizes studies of the role of SCF and its active ingredients in protecting against NDs, and highlights them as promising resources for future treatment. Furthermore, novel insights on the future challenges of SCF and its active ingredients are offered.

## 1. Introduction

Neurological diseases (NDs) are a major public health problem, with high prevalence, and leading to disability and mortality. The World Health Organization estimates that NDs and their sequelae affect as many as one billion people worldwide and are major factors contributing to associated disability and suffering. Cerebrovascular diseases, neurodegenerative diseases, and mental disorders, such as stroke and dementia, rank among the leading causes of death and disability, often affecting the adults in working-age [1]. The health index level of NDs is closely related to the level of regional socioeconomic development. In low- and middle-income countries, the prognosis of NDs is worse, as the resources to treat and manage patients are limited [2]. In China, the prevalence of cerebrovascular diseases has increased to 12.3‰ in rural areas, as evidenced by a survey taken every five years, from 1993 to 2013 [3]. The current number of cardiovascular and cerebrovascular diseases patients is 290 million, including 13 million stroke patients.

The treatment of NDs, including stroke and Alzheimer’s disease (AD), is critical to patients’ lifespan and quality of life. However, effective therapeutic drugs and methods are very limited. Even in high-income countries, stroke remains a common cause of death and disability [4], and women experience more stroke over their lifetime and more deaths from stroke [5,6], compared with men. The management of patients who suffer from acute ischemic stroke at an early stage is crucial and existing drugs are limited [7]. In addition, AD is a progressive neurodegenerative phenotype with complex cerebrovascular disorders [8]. The current treatment of AD mainly consists of neuroleptics, antidepressants, and benzodiazepines. However, drug interactions and toxicity resulting from the long-time use of pleiotropic drugs exacerbate the clinical symptoms of patients [9]. Therefore, it is necessary to find effective drugs to treat these NDs.

*Schisandra chinensis* Fructus (SCF) is the dry ripe fruit of *Schisandra chinensis* (Turcz.) Baill, which tastes sweet and sour. In traditional Chinese medicine it is mainly used for the treatment of dysphoria and palpitation, insomnia, and many dreams resulting from the poor preservation of the patient’s spirit [10,11]. The main components of SCF include lignans, volatile oils, and polysaccharides [12,13]. Previous studies have revealed the properties of SCF and its active components, including anti-myocardial dysfunction [14], anti-myocardial ischemia/reperfusion (I/R) injury [15], hepatoprotective effects [16], anti-tumor effects [17], and anti-HIV effects [18]. More recent advances have demonstrated that SCF and its active ingredients, schizandrin A (Sch A), schizandrin B (Sch B), schizandrin C (Sch C), schisantherin A (STA), schisandrin (SCH), schizandrol B, α-isocubebenol (ICO), gomisin A, gomisin N, and nigranoic acid, manifest protective effects on hypoxia-ischemia neural injury and neurodegenerative diseases, including stroke, AD, and Parkinson’s disease (PD). This paper summarizes the neuroprotective effects of SCF and its active ingredients, and provides a reference for the treatment of NDs.

## 2. Literature and Data Search Methodology

Pathway and biological term enrichment was based on the Bioinformatics Analysis Tool for Molecular mechANism of Traditional Chinese Medicine (BATMAN-TCM) [19]. The literature search was based on electronic databases, including PubMed/MEDLINE, CNKI, ScienceDirect, and Scopus, from 2000 to 2018. Search terms included SCF, SCF ingredients, SCF lignans, NDs, cerebrovascular diseases, neurodegenerative diseases, neuron, brain, oxidative stress, apoptosis, inflammation, neurotransmitters disorders, stroke, AD, PD, depression, and anxiety.

## 3. Biological Function Enrichment of SCF

**Figure 1:**
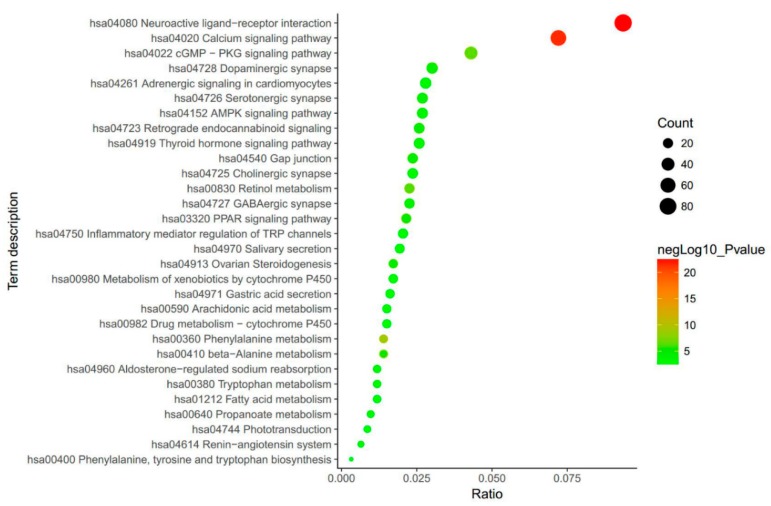
The biological mechanisms enrichment of *Schisandra chinensis* Fructus (SCF). The round represents the relationship between SCF and the biological terms. The size of the round signifies the count of the signaling pathways or functions. The color denotes the log10 of *p* value. The closer to red, the smaller *p* value is.

The results from searching in the BATMAN database showed that the biological mechanisms of SCF are mostly linked to neurologically related functions (Figure 1). Of the top 15 biological terms, 11 are strongly linked to mental functions, namely, neuroactive ligand–receptor interaction, the calcium signaling pathway, the cGMP-dependent protein kinase (cGMP-PKG) signaling pathway, dopaminergic synapse, serotonergic synapse, the adenosine monophosphate activated protein Kinase (AMPK) signaling pathway, retrograde end cannabinoid signaling, gap junctions, cholinergic synapses, the peroxisome proliferators activated receptor (PPAR) signaling pathway, and inflammatory mediator regulation of transient receptor potential (TRP) channels. The results indicated that SCF and its bioactive ingredients could potentially be treatments for NDs.

A literature search was carried out, focusing on the protective effect of SCF and its active components on NDs. The results showed that their main mechanisms are antioxidation, suppression of apoptosis, anti-inflammation, regulation of neurotransmitters, and modulation of pathways related to brain-derived neurotrophic factor (BDNF) (Table 1).

## 4. Antioxidative Effect in Neurological Diseases

Oxidative stress is one of the main causes of neural injury and neurodegeneration [54]. Moreover, because of the fact that antioxidant substances cannot easily penetrate the blood–brain barrier, brain tissue is particularly sensitive to oxidative stress [55]. The oxidizable/reducible chemical pairs, including reduced thioredoxin/oxidized thioredoxin, glutathione/glutathione disulfide, and NAD^+^/NADH (and NADP/NADPH), determine the overall redox potential of a cell [56].

**Figure 2:**
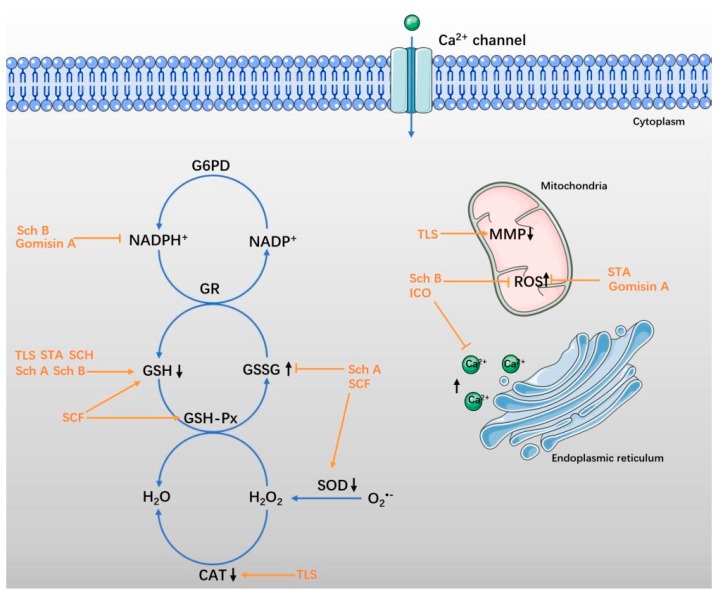
SCF and its active ingredients protect against oxidative stress in neurological diseases (NDs). Under pathological conditions, the redox balance is disrupted. The degradation of glutathione (GSH) is accelerated when the GSH-Px activity is decreased, and the production of glutathione disulfide (GSSG) is increased [64,65,66]. The expression of enzymes with antioxidant effects, as superoxide dismutase (SOD) and catalase (CAT), are inhibited simultaneously [67,68,69]. The mitochondrial membrane potential (MMP) decreases, while reactive oxygen species (ROS) is released excessively [70,71]. Intracellular Ca^2+^ influx, as well as intracellular Ca^2+^ release from the endoplasmic reticulum are increased, resulting in a series of downstream pathological responses [72,73,74]. The protective effect of SCF and its active ingredients are shown in orange.

Increasing evidence demonstrates that oxidative stress participates in the pathophysiological processes of stroke (including ischemia-reperfusion injury) and other brain injuries [57,58]. The production of reactive oxygen species (ROS) rapidly increases and overwhelms the antioxidant defenses. An excess of ROS directly modifies or degenerates cellular macromolecules, causing lipid peroxidation, protein oxidation, and DNA damage in neural tissues, and finally leading to brain injury [59,60]. In neurodegenerative diseases, the increased ROS leads to neuronal dysfunction. In the early events of AD, ROS are related to Aβ-induced nerve injury, as well as the abnormal phosphorylation of tau proteins. In addition, the accumulated ROS exacerbate dopaminergic neuronal death in the substantia nigra of PD patients [61]. In neuronal excitotoxicity, stroke, and neurodegenerative disease, increased extracellular glutamate levels bring about calcium overload, as well as mitochondrial dysfunction [62]. Therefore, redox regulation has recently been recognized as an important factor in acute and chronic NDs [63]. SCF and its ingredients were shown to manifest neuroprotective effects on NDs by attenuating oxidative stress (Figure 2). The pharmacological data are shown in Table 2.

### 4.1. SCF and Total Lignans of SCF

SCF was supposed to be a complementary medicine in cyclophosphamide (CTX) treatment for its effect of reducing chloroacetaldehyde (CAA) production and decreasing the C_max_ and AUC0-24h of 2-dechloroethylcyclophosphamide (DCCTX). With SCF treatment, brain glutathione (GSH) content increased and malondialdehyde (MDA) levels were reduced in rats with CTX-induced damage [20]. Yang et al. reported that SCF showed an antioxidant effect on AD rats by elevating superoxide dismutase (SOD) and glutathione peroxidase (GSH-Px) activity, and reducing MDA level [21].

The lignans extracted from SCF were identified as a potential treatment for AD, because of their protection against damage from oxidative stress. In a recent report, the total lignans of SCF (TLS) blocked the decrease of mitochondrial membrane potential (MMP) in primary mouse neuronal cells. Moreover, TLS restored the activity of total antioxidant capacity (T-AOC) in AD mice (see Section 5.1 and Section 6.1 for more detail) [26]. In addition, the lignans of SCF were assumed to protect against D-galactose (D-gal)-induced neurotoxicity in rats by maintaining GSH, MDA, and nitric oxide (NO) levels, and alleviating the decrease of SOD, catalase (CAT), and T-AOC activity. They were demonstrated to be potential candidates for the treatment of aging-associated neurodegenerative diseases [27].

### 4.2. Sch A and Sch B

Sch A and Sch B, derived from SCF, manifested anti-oxidative effects on AD. In research by Hu et al. Sch A significantly attenuated short-term and spatial memory impairments in AD mice by upregulating SOD, MDA, GSH-Px, GSH levels, and glutathione disulfide (GSSG) levels [30]. Furthermore, Sch B attenuated learning and memory impairment of AD mice induced by Aβ1-42. The restoration of glutamate transporter type 1 (GLT-1) and the capacity of glycogen synthase kinase3β (GSK3β) were maintained by Sch B treatment [35].

In a study by Chen et al. Sch B showed a protective effect in rats with cerebral ischemia/reperfusion (I/R) injury by strengthening the cerebral mitochondrial antioxidant effect. With the Sch B treatment, the GSH, α-TOC, and Mn-SOD expressions were increased, whereas the MDA-level and Ca^2+^-induced permeability transition was decreased [34]. In addition, Sch B relieved microglial-mediated inflammatory injury by inhibiting ROS and NADPH oxidase activity (see Section 6.2 for more detail) [36]. Sch B also modulated acetylcholine (ACh) activity in mice with dementia induced by scopolamine. The ACh level was maintained as normal, while the acetylcholinesterase (AChE) activity was inhibited by Sch B [33].

### 4.3. STA and SCH

STA is regarded as a neuroprotective lignin and works by attenuating the damage induced by 6-hydroxydopamine (6-OHDA) during in vivo and in vitro experiments. It alleviated neural damage by inhibiting ROS and NOS overproduction, and regulating extracellular signal-regulated kinase (ERK) phosphorylation, phosphatidylinositol 3-kinase (PI3K)/Akt ratio, and GSK3β dephosphorylation [42]. Moreover, STA restored SOD, GSH-Px, MDA, and GSH activity in AD mice, which indicated its protective effect against cognitive deficits and oxidative stress [43].

SCH is a bioactive lignan isolated from SCF. It has been suggested as a potential cognitive enhancer against AD through an antioxidative effect. As Hu et al. reported, SCH improved short-term and spatial memory impairments by upregulating SOD, GSH-Px, and GSH activity, and downregulating MDA and GSSG levels in the cerebral cortex and hippocampus of AD mice [45].

### 4.4. ICO and Gomisin A

ICO isolated from SCF showed an antioxidative effect on 6-OHDA-induced human neuroblastoma SH-SY5Y cell (a human derived cell line used as in vitro models of neuronal function and differentiation) death, inhibiting ROS and calcium accumulation. Additionally, ICO stimulated the expression of the antioxidant response genes NQO1 and HO-1 (see Section 5.4 for more detail) [47]. Moreover, gomisin A inhibited the ROS production, NADPH oxidase activation, and gp91phox expression induced by lipopolysaccharide (LPS) in microglia (see Section 6.3 for more detail) [50].

## 5. Suppression of Apoptosis

**Figure 3:**
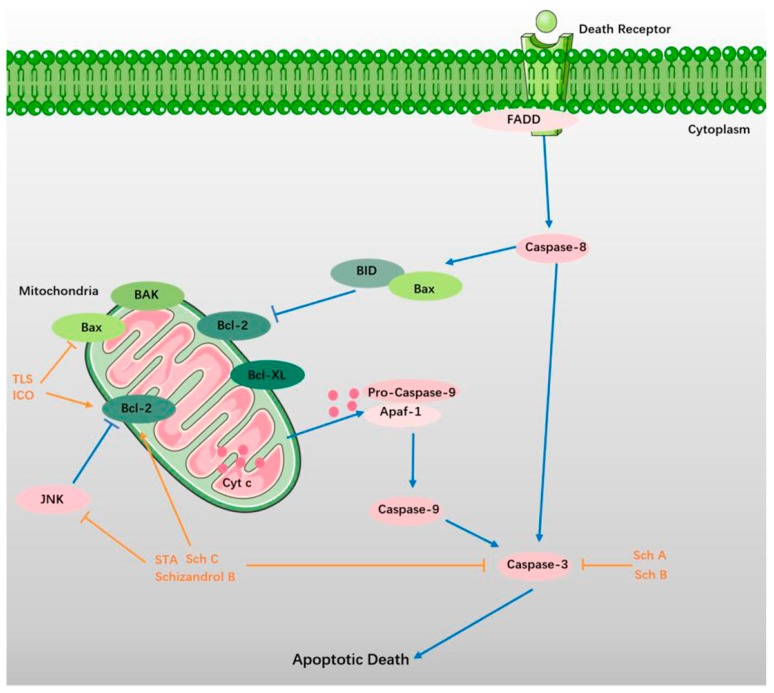
SCF and its ingredients attenuate apoptosis in NDs. Apoptosis are initiated by various external factors through the signal transduction of apoptosis signal with membrane receptors [83,84]. The apoptosis-inducing complex on the cell membrane includes a Fas-assiociated protein with death domain protein (FADD), of which N-terminal (DED) homophilic crosslinks with the inactive caspase-8. With the activating of caspase-8, the following cascade reactions are promoted [85,86,87]. Bax migrates from the cytosol to the mitochondria in apoptosis [88,89]. Mitochondrial Bcl-2 exerts an anti-apoptotic effect by preventing the release of mitochondrial cytochrome c (Cyt c), and reducing the activity of caspase [90,91,92]. Cyt c released into the cytoplasm binds to apoptosis-related factor 1 (Apaf-1) in the presence of dATP, and forms apoptotic bodies with caspase-9. With the activating of caspase-9, caspase-3 is subsequently activated to induce apoptosis [93,94,95]. The protective effect of SCF and its active ingredients are shown in orange.

Apoptosis is the main mechanism behind the appearance of DNA in circulation [75]. On the one hand, apoptosis may contribute to a significant proportion of neuronal death following acute brain ischemia (ABI), which may lead to stroke [76]. On the other hand, when ischemic stroke and neurodegenerative diseases such as AD and PD occur, the apoptosis results in profound brain injury, including neuronal death and loss of neurological functions [77,78,79]. More recent advances have revealed that the cell death pathways of apoptosis, intracellular Ca^2+^ homeostasis, and key metabolic pathways are regulated by mitochondria in neurologic disease [80]. More specifically, with more suppressed mitochondrial respiration comes more dysregulated calcium signaling. Furthermore, caspase-dependent and apoptosis-inducing factor-dependent apoptotic cell deaths are activated by Bax-dependent mitochondrial permeabilization [81,82]. SCF and its ingredients protect against NDs by suppressing apoptosis (Figure 3). The pharmacological data are shown in Table 3.

### 5.1. TLS

In a study by Jiang et al. TLS manifested a protective effect on rats with cerebral ischemia injury. The mechanism is related to increased Bcl-2 and p-Akt levels and the inhibition of apoptin Bax expression in the cerebral infarction area [28]. Moreover, TLS showed significant antiapoptotic effects in Aβ1–42-induced AD in primary mouse neuronal cells, by increasing Bcl-2 expression [26].

### 5.2. Sch A and Sch B

Sch A has been reported to reduce cell apoptosis and necrosis in primary cultures of rat cortical neurons after oxygen and glucose deprivation, followed by reperfusion (OGD/R). Intracellular Ca^2+^ and LDH levels were decreased by Sch A treatment. Proteins play an important role in neuronal apoptosis, c-Jun NH2-terminal kinases (JNK), p38, and caspase-3 were modulated by Sch A in H293T cells [31]. Furthermore, Sch B showed antiapoptotic and anti-autophagy effects in rats with AD induced by Aβ (1–40). In these experiments, the overexpression of caspase-3 and terminal transferase-mediated dUTP nick-end labeling (TUNEL) positive cells were suppressed by Sch B treatment. In addition, proteins such as HSP70 and beclin-1 were upregulated by Sch B (see Section 6.2 for more detail) [37].

### 5.3. STA, Sch C, and Schizandrol B

As Sa et al. reported, STA pretreatment inhibited 1-methyl-4-phenylpyridinium ion (MPP^+^)-induced cytotoxicity in SH-SY5Y cells and 1-methyl-4-phenyl-1,2,3,6-tetrahydropyridine (MPTP)-induced the loss of TH-positive dopaminergic neurons in PD mice. The mechanism was suggested to increase cAMP-response element binding protein (CREB)-mediated Bcl-2 expression and activate PI3K/Akt signaling [44]. In addition, STA, Sch C, and Schizandrol B showed beneficial effects in preventing serum and glucose deprivation (SGD) injury. Overexpressed proteins related to apoptosis were regulated by these lignans [40].

### 5.4. ICO and Gomisin A

α-Isocubebenol (ICO) derived from SCF was recently shown to exert neuroprotective properties with an antiapoptotic effect. In the scopolamine-induced AD mice, ICO significantly upregulated the Bcl-2/Bax ratio. In addition, the AChE activity and decreased ERK phosphorylation induced by scopolamine were attenuated by ICO treatment [48]. In an in vitro experiment, ICO showed a protective effect on 6-OHDA-induced neural damage in SH-SY5Y cells. The mechanism was suggested to inhibit the release of the apoptosis-inducing factor from the mitochondria into the cytosol and nucleus [47]. In addition, gomisin A protected against CTX toxicity by blocking CYP3A-mediated metabolism and reducing CAA production in GH3 cells [51].

## 6. Anti-Inflammatory Effect

**Figure 4:**
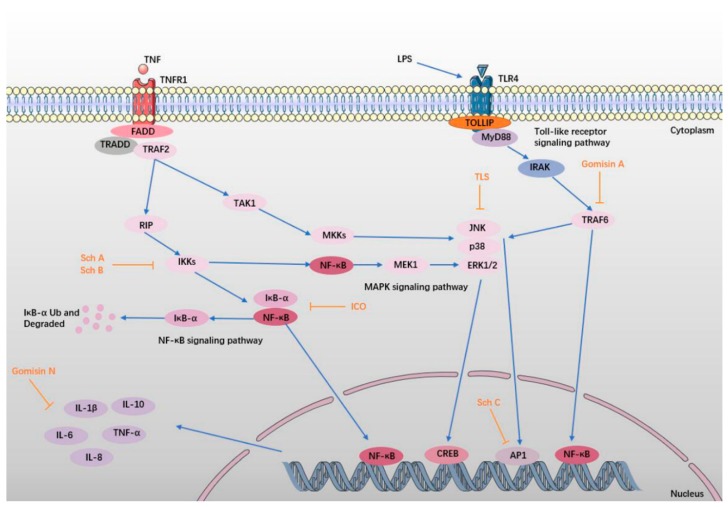
SCF and its active ingredients protect against inflammation in NDs. In the inflammatory response, TLR4 recognizes lipopolysaccharide (LPS), and then binds to the MyD88 Toll structure, forming a TLR-MyD active complex. Then, the complex recruits and activates the IL-1 receptor-associated kinase (IRAK), which is associated with tumor necrosis factor-associated factor 6 (TRAF6), activating the downstream mitogen-activated protein kinases (MAPK) pathway [101,102]. Meanwhile, TNFR1 binds to TNF, and interacts with receptor-interacting protein (RIP), activating the downstream inhibitory kappa B kinase (IKK) and MAPK pathway [103,104]. Phosphorylation of IκB protein leads to degradation of the protein, promotes nuclear translocation of nuclear factor-κB (NF-κB), and transfers NF-κB to the nucleus [105]. At the same time, the activation of the MAPK pathway leads to the production of activator protein-1 (AP-1), which is phosphorylated, and then enters the nucleus. Activation of NF-κB and AP-1 can lead to over-expression of the inflammatory factors, such as TNF-α, IL-1β, IL-6, IL-8, and IL-10, resulting a series of inflammatory reactions [106,107,108,109]. The protective effect of SCF and its active ingredients are shown in orange.

Neuroinflammation has been proven to contribute to the etiology of hypoxia-ischemia neural injury and neurodegenerative diseases [96]. Despite discrepancies in their pathophysiological timeframe and severity, NDs share common molecular mechanisms that include inflammation, mitochondrial dysfunction, and endoplasmic reticulum stress [79]. In an ischemic stroke, neuroinflammatory processes are upregulated and initiate a feedback loop of inflammatory cascades that can expand the region of damage [97]. Inflammatory molecules such as cytokines, chemokines, and reactive oxygen and nitrogen species are thought to be pivotal mediators of persistent neuronal injury [98,99,100]. SCF and its ingredients exert a neuroprotective effect on NDs by alleviating inflammation (Figure 4). The pharmacological data are shown in Table 4.

### 6.1. TLS

As Zhao et al. reported, TLS protects against cognitive deficits and neurodegeneration by inhibiting the expression of JNK/p38 and BACE1 in Aβ1–42-induced primary mouse neuronal cells. These results indicated that TLS could be applied as an active pharmaceutical ingredient for cognitive improvement in AD [26]. Furthermore, the lignans isolated from SCF, including Sch A–D, manifested beneficial activity by inhibiting the lipopolysaccharide (LPS)-induced NO release in primary murine BV2 microglia cells [29].

### 6.2. Sch A, Sch B, and Sch C

Song et al. reported that Sch A can exert anti-inflammatory and neuroprotective effects on LPS-induced inflammatory injury in microglia (BV2 cells) and neurons. The potential molecular mechanism may be the inhibition of the tumor necrosis factor-associated factor 6(TRAF6)- inhibitory kappa B kinase (IKK)β/ nuclear translocation of nuclear factor-κB (NF-κB) and Janus kinase-2/signal transducer and activator of transcription-3 (Jak2/Stat3) signaling pathways [32].

Sch B has been effective at inhibiting neural inflammation during in vivo and in vitro studies. Giridharan reported that Sch B modulated receptors for advanced glycation end products (RAGE), NF-κB, and the mitogen-activated protein kinases (MAPK) signaling pathway. Moreover, an overexpression of the proteins prompting inflammation were inhibited by Sch B [37]. As Lee reported, Sch B attenuated cerebral ischemia injury in rats by suppressing the overexpression of inflammatory markers in ischemic hemispheres [39], and relieved microglial-mediated inflammatory injury by inhibiting the TLR4-dependent MyD88/IKK/NF-κB signaling pathway [36]. Moreover, Sch B showed an inhibitory effect on the LPS-induced inflammatory response by suppressing NF-κB activation, while activating PPAR-γ [38].

As Park et al. reported, Sch C was regarded as a natural antineuroinflammatory agent, protecting against lipoteichoic acid (LTA)-stimulated inflammation in mouse primary microglia. The results showed that Sch C suppressed NF-κB, AP-1, JAK-STATs, and MAPK expression, and activated cAMP/PKA/CREB and Nrf-2 signaling [41].

### 6.3. ICO, Gomisin A, and Gomisin N

ICO showed a protective effect on Aβ-stimulated neuroinflammation in mouse primary microglia. The research indicated that ICO provided a neuroprotective function by inhibiting IκB-α, NF-κB, and the MAPK signaling pathway [49].

As one of the major dibenzocyclooctadiene lignans isolated from SCF, gomisin A manifested as a neuroprotective treatment for LPS-stimulated inflammation on N9 microglia. The potential mechanism of gomisin A was suggested to be inhibition of the TLR4-mediated NF-κB and MAPKs signaling pathways [50]. As Araki et al. reported, gomisin N ameliorated LPS-induced inflammation in mice and BV2 cells. The research demonstrated that an elevation of the inflammatory markers induced by LPS was inhibited by gomisin N treatment [52].

## 7. Regulation of Neurotransmitters

The emotional processing and behavioral anxiety are determined by the reciprocal relationship between the central nervous system and the endocrine signals. Peptide hormones are increasingly recognized for their effects on anxiety-like behavior and reward [110]. The neurobiological bases of depression and anxiety disorders are not fully understood and the currently available treatments are not always effective [111]. In recent years, the disorders of neurotransmitters, including norepinephrine (NE), 5-hydroxytryptamine (5-HT), dopamine (DA), and gamma-aminobutyric acid (GABA) have been reported to lead to significant changes in neurodegenerative diseases and induce anxiety, depression, arousal, and alarm [112,113,114]. They are involved in the pathophysiological bases of these diseases and provide benefits in their treatment through their diverse functions [115,116]. Despite this, antidepressant and anxiolytic drug development has largely stalled [117].

SCF was demonstrated to ameliorate 4-chloro-dl-phenylalanine (PCPA) induced insomnia in rats by regulating the expression of brain neurotransmitters and their metabolites through its sedative-hypnotic effects [25]. Furthermore, SCF was used as an efficient treatment for anxiety-like behavior induced by ethanol withdrawal. The results showed that it attenuated anxiety by significantly downregulating the elevation of norepinephrine (NE) and its metabolite in the hypothalamic paraventricular nucleus [24]. According to the latest report, SCH showed a neuroprotective effect by ameliorating learning and memory impairments in APP/PSI transgenic mice. The mechanism was suggested to be regulation of neurotransmitters and their metabolites in the brain. The results indicated that SCH could be applied as an active pharmaceutical compound for neurodegenerative diseases such as PD and AD [46]. The pharmacological data are shown in Table 5.

## 8. Modulation of BDNF Related Pathways

As a growth factor dynamically expressed in the brain across postnatal development, BDNF regulates neuronal differentiation and synaptic plasticity. It is acknowledged that decreased BDNF levels lead to altered neural plasticity, contributing to disease [118]. The mechanism of BDNF release appears to be related to synaptic sprouting and strengthened synaptic connections [119]. Nowadays, depression and anxiety are becoming major burdens to society, affecting as much as 7% of the world’s population [120]. BDNF has been introduced to treatment-resistant depression and it has been identified as a therapeutic target for depression [121,122]. Furthermore, it is a distinct marker of stress adaptation, extinction of fear, and neuroimmune response [123,124,125].

Yan et al. reported that SCF could improve a depression-like emotional state and associated cognitive deficits in mice with chronic unpredictable mild stress (CUMS). The mechanism was proven to regulate BDNF expression in the hippocampus as well as upregulate the TrkB/CREB/ERK and PI3K/Akt/GSK-3β pathways [22,23]. Moreover, Yuan et al. reported that nigranoic acid (SBB1, 3,4-secocycloartene triterpenoid) manifested beneficial effects in terms of enhancing mental and intellectual functions by increasing BDNF and c-fos expression in NGF-differentiated PC12 cells [53]. The pharmacological data are shown in Table 6.

## 9. Conclusions and Perspectives for Future Work

SCF and its active ingredients manifest a protective effect on NDs by attenuating injury induced by overoxidative stress, apoptosis, inflammation, and neurotransmitter disorders. The most active ingredients in SCF, lignans, share the same physiologically active structure as biphenyl cycloalkenol, whose parent nucleus is biphenyl cyclooctadiene [126]. Biphenyl cyclooctadiene has a biphenyl structure, as well as the eight-membered ring structure of biphenyl and side-chain synthesis. Given its many structural forms and stereoisomers, it is acknowledged as the key structure displaying antioxidation, antiapoptosis, and antiviral effects [127]. In future studies, attention should be paid to the components of the key active structures, so as to screen out lead compounds. The structure of the lead compounds should be optimized to enhance metabolic stability and improve bioavailability, in order to provide new candidates for the clinical treatment of NDs.

The pathogenesis of NDs has been further elucidated in recent years, such as the mitochondrial mechanism of neuroglial crosstalk after stroke [128], phagocytosis of reactive astrocytes following brain ischemia [129], purinergic signaling in reactive astrocytes of AD [130], endothelial cytoskeletal reorganization in blood–brain barrier disruption [131], cerebral cavernous malformations in stroke, and seizure [132,133]. Furthermore, more therapeutic targets of NDs have been discovered recently, such as TRPA1 [134], IL-27 [135], TIM-3 [136], tau [137], and histamine H3 receptor [138]. As biologically active drugs, in future work, SCF and its active ingredients should be applied to many more target-screening models.

## Figures and Tables

**Table 1:** Summary of the pharmacological effects and biological analysis of *Schisandra chinensis* Fructus (SCF) and its active ingredients. BDNF—brain-derived neurotrophic factor; CREB—cAMP-response element binding protein; PI3K—phosphatidylinositol 3-kinase; GSK—glycogen synthase kinase; TLS—total lignans of SCF; SCH—schisandrin; ICO—α-isocubebenol; STA—schisantherin A; GSH—glutathione; NO—nitric oxide; ERK—extracellular signal-regulated kinase; NE—norepinephrine; MAPK—mitogen-activated protein kinases; TRAF6—tumor necrosis factor-associated factor 6; IKK—inhibitory kappa B kinase; NF-κB—nuclear translocation of nuclear factor-κB; Jak2/Stat3—Janus kinase-2/signal transducer and activator of transcription-3; GLT-1—glutamate transporter type 1; NADPH—nicotinamide adenine dinucleotide phosphate; JNK—c-Jun NH2-terminal kinases; RAGE—receptors for advanced glycation end products; ROS—reactive oxygen species.

SCF and Its Active Ingredients	Pharmacological Activity	Biological Analysis	Key References
SCF	Anti-oxidant	GSH antioxidant response	[20,21]
	Modulate BDNF related pathways	BDNF, TrkB/CREB/ERK and PI3K/Akt/GSK-3β pathways	[22,23]
	Regulate neurotransmitters	NE activity	[24]
		Neurotransmitters activities	[25]
TLS	Anti-oxidant	Mitochondrial function	[26]
		GSH antioxidant response	[27]
	Anti-apoptosis	Bcl-2 expression	[26]
		Bcl-2 and Bax expression	[28]
	Anti-inflammatory	NO activity	[29]
		MAPKs signaling	[26]
Sch A	Anti-oxidant	GSH antioxidant response	[30]
	Anti-apoptosis	ERK, JNK, Caspase-3 signaling	[31]
	Anti-inflammatory	TRAF6/IKKβ/NF-κB and Jak2/Stat3 signaling pathways	[32]
Sch B	Anti-oxidant	ACh activity	[33]
		GSH antioxidant response	[34]
		GLT-1 and GSK3β activities	[35]
		ROS, NADPH oxidase activity	[36]
	Anti-apoptosis	Caspase-3, HSP70, beclin-1 expression	[37]
	Anti-inflammatory	RAGE, NF-κB, MAPKs signaling	[37]
		PPAR-γ activity	[38]
		MyD88/IKK/NF-κB signaling pathway	[36]
		TNF-α, IL-1β activities	[39]
Sch C	Anti-apoptosis	JNK/Caspase-3 signaling	[40]
	Anti-inflammatory	cAMP/PKA/CREB and Nrf-2 signaling	[41]
STA	Anti-oxidant	MAPKs, PI3K/Akt and GSK3β signaling	[42]
		GSH antioxidant response	[43]
	Anti-apoptosis	Bcl-2 expression and PI3K/Akt signaling	[44]
		JNK/Caspase-3 signaling	[40]
SCH	Anti-oxidant	GSH antioxidant response	[45]
	Regulate neurotransmitters	Neurotransmitters and their metabolites effects	[46]
Schizandrol B	Anti-apoptosis	JNK/Caspase-3 signaling	[40]
ICO	Anti-oxidant	ROS and calcium accumulation	[47]
	Anti-apoptosis	CREB/Nrf-2 signaling	[47]
		Bcl-2 and Bax expression	[48]
	Anti-inflammatory	NF-κB and MAPK signaling pathways	[49]
Gomisin A	Anti-oxidant	ROS, NADPH oxidase activity	[50]
	Anti-apoptosis	CYP3A activity	[51]
	Anti-inflammatory	TLR4 mediated NF-κB and MAPKs pathways	[50]
Gomisin N	Anti-inflammatory	Inflammatory responses and neural activation	[52]
Nigranoic acid	Modulate BDNF related pathways	ERK1/2, Ca^2+^-CaMKII pathways, BDNF activity	[53]

**Table 2:** The pharmacological data of SCF and its active ingredients in protecting against NDs by anti-oxidative effect. LPS—lipopolysaccharide; 6-OHDA—6-hydroxydopamine; CTX—cyclophosphamide; AD—Alzheimer’s disease; NS—neurological disease; MDA—malondialdehyde; I/R—ischemia/reperfusion; T-AOC—total antioxidant capacity; GSSG—glutathione disulfide; CAT—catalase.

SCF and Its Active Ingredients	Study Design	Study Type	Molecular and Cellular Mechanisms of Action	Dose Range	Minimal Active Concentration	Key Reference
SCF	CTX induced brain injury in rats	In vivo	Increases GSH content	0.10–1.00 g/kg	0.50 g/kg	[20]
			Decreases MDA levels			
	intra-hippocampal Aβ1-42 induced AD in rats	In vivo	Increases SOD and GSH-Px activity	200 mg/kg	200 mg/kg	[21]
TLS	Aβ1-42 induced AD in primary mouse neuronal cells	In vitro	Blocking the decrease of MMP	10, 30, 100 μM	10 μM	[26]
	Aβ1-42 induced AD in mice	In vivo	Restroes T-AOC and MDA level	50, 200 mg/kg	50 mg/kg	
			Ameliorates the neurodegeneration in the hippocampus			
	D-galactose (D-gal)-induced neurotoxicity in rats	In vivo	Attenuates SOD, CAT, T-AOC decreasing	―	―	[27]
			Maintains GSH, MDA, NO levels			
Sch A	Aβ1-42 induced AD in mice	In vivo	Increases SOD, GSH-Px, GSH levels	4, 12, 36 mg/kg	12 mg/kg	[30]
			Decreases MDA, GSSG levels			
Sch B	SP induced dementia in mice	In vivo	Suppresses AChE (acetylcholinesterase) activity	10, 25, 50 mg/kg	25 mg/kg	[33]
			Maintaines ACh level			
	Occlusion (using aneurysm clips) induced cerebral I/R injury	In vivo	Increases GSH, α-TOC, Mn-SOD	1, 10, 30 mg/kg	1 mg/kg	[34]
			Decreases MDA, Ca^2+^, MPT			
	Aβ1-42 induced AD in mice	In vivo	Restroes GLT-1 and GSK3β activities	0.15 mg/kg		[35]
			Decreases hyperphosphorylated tau protein			
	Microglial-mediated inflammatory injury	In vitro	Inhibites ROS, NADPH oxidase activity	5, 10, 20 μM	5 μM	[36]
STA	6-OHDA-induced neural damage in SH-SY5Y cells	In vitro	Decreases cytotoxicity	3, 6, 12, 25, 50, 100 μM	14.8 μM (EC50)	[42]
			Down-regulates ROS level			
			Inhibites NO, iNOS levels			
			Opposes ERK phosphorylation decreases			
			Up-ragulates p-Akt/t-Akt ratio			
			Preventes GSK3β dephosphorylation			
	6-OHDA-induced neural damage in zebrafish	In vivo	Prevents dopaminergic neuron loss	2.5, 5, 10 μM	10 μM	
	Aβ1-42 induced AD in mice	In vivo	Restroes SOD, GSH-Px, MDA, GSH activites	0.01–0.1 mg/kg	0.1 mg/kg	[43]
SCH	Aβ1-42 induced AD in mice	In vivo	Increases SOD, GSH-Px, GSH levels	4, 12, 36 mg/kg	36 mg/kg	[45]
			Decreases MDA, GSSG levels			
ICO	6-OHDA-induced neural damage in SH-SY5Y cells	In vitro	Inhibites ROS	20, 40, 80 µM	40 µM	[47]
			Inhibites calcuim accumulation			
			Increases NQO1, HO-1 levels			
Gomisin A	LPS-stimulated N9 microglia	In vitro	Inhibites ROS, NADPH, gp91phox expression	1–100 µM	3 µM	[50]

**Table 3:** The pharmacological data of SCF and its active ingredients in protecting against NDs by suppressing apoptosis. TUNEL—terminal transferase-mediated dUTP nick-end labeling; OGD/R— oxygen and glucose deprivation followed by reperfusion.

SCF and Its Active Ingredients	Study Design	Study Type	Molecular and Cellular Mechanisms of Action	Dose Range	Minimal Active Concentration	Key Reference
TLS	Aβ1-42 induced AD in primary mouse neuronal cells	In vitro	Increase Bcl-2 expressions	10, 30, 100 μM	10 μM	[26]
	Suture-occluded induced cerebral ischemia injury	In vivo	Inhibites Bax level	25–100 mg/kg	25 mg/kg	[28]
			Increases Bcl-2, p-Akt levles			
Sch A	OGD/R-induced cell death in primary culture of rat cortical neurons	In vitro	Decreases Ca^2+^, LDH levels	1.25, 2.5, 5 μg/mL	1.25 μg/mL	[31]
			Up-regulates C3aR, C5aR levels			
	H293T cell		Down-regulates ERK, JNK, p38, caspase-3 levels			
Sch B	Aβ-induced neuronal dysfunction in rats	In vivo	Inhibites Caspase-3, TUNEL positive cells	25 or 50 mg/kg	25 mg/kg	[37]
			Up-regulates HSP70, beclin-1			
Sch C, Schizandrol B	Serum and glucose deprivation (SGD) injury in SH-SY5Y cells	In vitro	Inhibites LDH level	2.5, 5.0 mg/mL	2.5 mg/mL	[40]
			Inhibites NLRP3, Caspase-1, IL-1β, NF-κB, plκB/lκB, pJNK1/2, JNK1/2, Caspase-3 expression			
STA	MPP^+^ induced neural damage in SH-SY5Y cells	In vitro	Decreases cytotoxicity	60 μM	60 μM	[44]
			Increases CREB, Bcl-2 expression			
			Activates PI3K and Akt levels			
	MPTP induced neural damage in mice (PD)	In vivo	Prevents TH-positive dopaminergic neurons loss	30, 100, 300 mg/kg	300 mg/kg	
	Serum and glucose deprivation (SGD) injury in SH-SY5Y cells	In vitro	Inhibites LDH level	2.5, 5.0 mg/mL	2.5 mg/mL	[40]
			Inhibites NLRP3, Caspase-1, IL-1β, NF-κB, plκB/lκB, pJNK1/2, JNK1/2, Caspase-3 expression			
ICO	6-OHDA-induced neural damage in SH-SY5Y cells	In vitro	Inhibites TUNEL positive cells	20, 40, 80 µM	40 µM	[47]
			Inhibites the release of AIF			
			Stimulates the activation of PKA/PKB/CREB/Nrf-2			
	SP induced memory impairment in mice (AD)	In vivo	Decreases AChE activity	5, 10 mg/kg	5 mg/kg	[48]
			Up-ragulates Bcl-2/Bax ratio			
			Attenuates the decrease of ERK phosphorylation			
Gomisin A	CTX induced brain injury in rats	In vivo	Blocking CYP3A-mediated metabolism	20.8 mg/kg	20.8 mg/kg	[51]
			Reducing CAA production			

**Table 4:** The pharmacological data of SCF and its active ingredients in protecting against NDs by anti-inflammation effect.

SCF and Its Active Ingredients	Study Design	Study Type	Molecular and Cellular Mechanisms of Action	Dose Range	Minimal Active Concentration	Key Reference
TLS	Aβ1-42 induced AD in primary mouse neuronal cells	In vitro	Decreases BACE1 activity	10, 30, 100 μM	10 μM	[26]
			Inhibites JNK/p38 expression			
	LPS-induced inflammation in microglia (BV2 cells)	In vitro	Inhibites NO level	1, 10 μM	10 μM	[29]
Sch A	LPS-induced inflammation in microglia (BV2 cells)	In vitro	Down-regulates the NO, TNF-α, IL-6 increasing	10, 20, 50 μM	10 μM	[32]
	Microglia-mediated inflammatory injury in neurons		Inhibites iNOS, COX-2 levels	10, 20, 50 μM	20 μM	
			Inhibites TRAF6-IKKβ-NF-κB pathway			
			Inhibites Jak2-Stat3 pathway activation and Stat3 nuclear translocation			
Sch B	Aβ-induced neuronal dysfunction in rats	In vivo	Inhibites iNOS, COX-2, IL-1β, IL-6, TNF-α levels and DNA damage	25 or 50 mg/kg	25 mg/kg	[37]
			Inhibites RAGE, NF-κB, MAPKs			
	LPS-induced inflammation in microglia (BV2 cells)	In vitro	Down-regulates TNF-α, IL-6, IL-1β, and PGE2 levels	12.5, 25, 50 μM	12.5 μM	[38]
			Inhibites NF-κB activation			
			Up-ragulates the expression of PPAR-γ			
	Microglial-mediated inflammatory injury	In vitro	Down-regulates NO, TNF-α, PGE2, IL-1β, IL-6 levels	5, 10, 20 μM	5 μM	[36]
			Inhibites TLR 4, MyD88, IRAK-1, TRAF-6 interaction			
			Inhibites IKK, NF-κB levels			
	Intraluminal thread induced focal cerbral ischemia in rats	In vivo	Inhibites TNF-α, IL-1β, matrix metalloproteinase (MMP)-2, MMP-9, OX-42 levels	10, 30 mg/kg	10 mg/kg	[39]
Sch C	LTA induced inflammation in mouse primary microglia	In vitro	Increases HO-1, NQO-1 levels	1, 5, 10, 20 μM	10 μM	[41]
			Activates cAMP, PKA, CREB, Nrf-2 levels			
			Attenuates ddAdo, H-89 levels			
			Inhibites PGE2, NO, ROS, iNOS, COX-2, MMP-9 expressions			
			Suppresses NF-κB, AP-1, JAK-STATs, MAPK activation			
ICO	Aβ-stimulated neuroinflammation in mouse primary microglia	In vitro	Inhibites PGE2, NO, ROS, MMP-9 levels	25, 50, 100 µM	100 µM	[49]
			Inhibites iNOS, COX-2 levels			
			Inhibites IκB-α, NF-κB, MAPK activities			
Gomisin A	LPS-stimulated inflammation N9 microglia	In vitro	Suppresses iNOS, COX-2 levels	1–100 µM	3 µM	[50]
			Attenuates TNF-α, IL-1β and IL-6 levels			
			Inhibited TAK1-IKKa/b-IκB -NF- κB and MAPKs inflammatory signaling pathways	30–100 µM	30 µM	
			Inhibited TLR4 expression			
Gomisin N	LPS-induced inflammatory and depressive symptoms in mice	In vivo	Inhibites iNOS, COX-2, IL-1β, IL-6, TNF-α levels	100 mg/kg	100 mg/kg	[52]
			Increases c-Fos immunopositive cells number			
	LPS-induced inflammation in microglia (BV2 cells)	In vitro	Inhibites iNOS, COX-2, IL-1β, IL-6, TNF-α levels	1.56–50 µM	25 µM	

**Table 5:** The pharmacological data of SCF and its active ingredients in protecting against NDs by regulating neurotransmitters. PCPA—4-chloro-dl-phenylalanine; GABA—gamma-aminobutyric acid; DA—dopamine.

SCF and its Active Ingredients	Study Design	Study Type	Molecular and Cellular Mechanisms of Action	Dose Range	Minimal Active Concentration	Key Reference
SCF	Ethanol withdrawal induced anxiety-like behavior	In vivo	Decreases NE and its metabolite			[24]
	PCPA induced insomnia in rat	In vivo	Reduces the elevation of GABA, NE, DA, DOPAC, HVA	7.5 g/kg	7.5 g/kg	[25]
			Increases 5-HT, 5-HIAA levels			
SCH	APP/PS1 transgenic mice (induced AD)	In vivo	Ameliorated the cognitive impairment	2 mg/kg	2 mg/kg	[46]
			Decreases Aβ deposition in the hippocampus			
			Regulates serotonin, 5-HIAA, DA, NE, γ-aminobutyric acid, glutamic acid, homovanillic acid, 3,4-dihydroxyphenylacetic acid and acetylcholine levels			

**Table 6:** The pharmacological data of SCF and its active ingredients in protecting against NDs by modulating BDNF related pathways. CUMS—chronic unpredictable mild stress.

SCF and Its Active Ingredients	Study Design	Study Type	Molecular and Cellular Mechanisms of Action	Dose Range	Minimal Active Concentration	Key Reference
SCF	Corticosterone induced depressive-like behavior in mice	In vivo	Up-ragulates BDNF/TrkB/CREB	300, 600 mg/kg	600 mg/kg	[22]
	CUMS-induced depression and cognitive impairment in mice	In vivo	Increases BDNF levels in hippocampus	600–1200 mg/kg	600 mg/kg	[23]
			Up-regulates TrkB/CREB/ERK			
			Up-regulates PI3K/Akt/GSK-3β			
Nigranoic acid	NGF-differentiated PC12 cells	In vitro	Increases BDNF, c-fos mRNA	1, 10, 50 µM	50 µM	[53]
			Increases cytoplasmic Ca^2+^, NO levels			
			Activates ERK1/2, CaMKII levels			
